# Visibility of Community Nursing Within an Administrative Health Classification System: Evaluation of Content Coverage

**DOI:** 10.2196/12847

**Published:** 2019-06-26

**Authors:** Lorraine J Block, Leanne M Currie, Nicholas R Hardiker, Gillian Strudwick

**Affiliations:** 1 School of Nursing University of British Columbia Vancouver, BC Canada; 2 School of Human and Health Sciences University of Huddersfield Huddersfield United Kingdom; 3 Campbell Family Mental Health Research Institute Centre for Addiction and Mental Health Toronto, ON Canada

**Keywords:** World Health Organization, classification, nursing informatics, medical informatics, data collection, terminology, community health services, standardized nursing terminology

## Abstract

**Background:**

The World Health Organization is in the process of developing an international administrative classification for health called the International Classification of Health Interventions (ICHI). The purpose of ICHI is to provide a tool for supporting intervention reporting and analysis at a global level for policy development and beyond. Nurses represent the largest resource carrying out clinical interventions in any health system. With the shift in nursing care from hospital to community settings in many countries, it is important to ensure that community nursing interventions are present in any international health information system. Thus, an investigation into the extent to which community nursing interventions were covered in ICHI was needed.

**Objective:**

The objectives of this study were to examine the extent to which International Classification for Nursing Practice (ICNP) community nursing interventions were represented in the ICHI administrative classification system, to identify themes related to gaps in coverage, and to support continued advancements in understanding the complexities of knowledge representation in standardized clinical terminologies and classifications.

**Methods:**

This descriptive study used a content mapping approach in 2 phases in 2018. A total of 187 nursing intervention codes were extracted from the ICNP Community Nursing Catalogue and mapped to ICHI. In phase 1, 2 coders completed independent mapping activities. In phase 2, the 2 coders compared each list and discussed concept matches until consensus on ICNP-ICHI match and on mapping relationship was reached.

**Results:**

The initial percentage agreement between the 2 coders was 47% (n=88), but reached 100% with consensus processes. After consensus was reached, 151 (81%) of the community nursing interventions resulted in an ICHI match. A total of 36 (19%) of community nursing interventions had no match to ICHI content. A total of 100 (53%) community nursing interventions resulted in a broader ICHI code, 9 (5%) resulted in a narrower ICHI code, and 42 (23%) were considered equivalent. ICNP concepts that were not represented in ICHI were thematically grouped into the categories family and caregivers, death and dying, and case management.

**Conclusions:**

Overall, the content mapping yielded similar results to other content mapping studies in nursing. However, it also found areas of missing concept coverage, difficulties with interterminology mapping, and further need to develop mapping methods.

## Introduction

### Introduction and Objectives

The digitalization of health care information is increasing rapidly. The use of standardized terminologies and classifications to unambiguously represent this information is a fundamental principle in the field of clinical and biomedical informatics [1]. The World Health Organization Family of International Classifications (WHO-FIC) contains a suite of standardized administrative classification products, which are used internationally and nationally to statistically report on the health and well-being of individuals, families, communities, and populations [2]. The WHO-FIC includes the International Classification of Diseases (ICD), the International Classification of Functioning, Disability and Health (ICF), and the International Classification of Health Interventions (ICHI; in development) [2].

ICHI is the newest classification of this group, and its purpose is to provide a common tool for reporting and analyzing health care interventions [3]. A series of international evaluative projects had been planned for the beta-1 release (eg, terminology mapping and standard case reporting) [4]. The goal of the evaluation projects was to ensure the terminology is (1) robust enough to capture interventions provided across the continuum, (2) appropriate to cover interventions provided by different health care disciplines, (3) has a functional browser tool, and (4) has the depth of educational and training material sufficient to support its future use [4]. Evaluations and releases of the ICHI beta version are ongoing, with a future goal of seeking World Health Assembly approval in 2019 [5].

This descriptive study represents an international evaluative project. Its objectives were to (1) examine the ability of ICHI to represent community nursing interventions found in the International Classification for Nursing Practice (ICNP), (2) provide recommendations for content development, and (3) support continued advancements in understanding the complexities of knowledge representation in standardized clinical terminologies and classifications. In this context, a community nursing intervention refers to the actions carried out by nurses practicing in a community setting to support the health and well-being of patients, families, communities, or populations [6-8]. The multiple research methods used to achieve these research objectives were based on a content mapping approach. Specifically, 2 clinical experts individually matched equivalent (or near equivalent) concepts from ICNP to ICHI. The results were compared and reviewed until matching consensus was reached between the 2 coders. This study is unique in that it is the first to bring a community nursing care perspective to the evaluation of ICHI, informing broader discussions about the representation of health care activities and resourcing in administrative classifications. To the best of our knowledge, it is also the first published study to evaluate aspects of the 2017 ICHI beta-1 release.

### Background

#### Community Nursing

With rapid population growth occurring worldwide, health care systems are challenged both socially and economically, with changing demographics, shifting disease patterns, increased prevalence of chronic diseases, and financial reforms [9]. The delivery of health care services outside of acute care centers is necessary to manage these complex phenomena. Therefore, community nursing is an essential global service. The WHO defines community nursing as a service which “combines the skills of nursing, public health and some phases of social assistance and functions as part of the total public health program for the promotion of health, the improvement of the conditions in the social and physical environment, rehabilitation of illness and disability” [10,11].

Nurses practicing in the community context provide care that directly improves the health outcomes of individuals, families, communities, and populations [12]. This can be attributed to the ethos of community nursing, where work is founded on the principles of social justice, holistic care, equity, ethics, community capacity building and empowerment, and action upon the intersectoral determinants of health [12]. The types of interventions community nurses provide are extensive and can include home visits for new baby and family care, school classes on the topic of sexual health, wound care, interventions that address preventing elder abuse, and advocacy for health and wellness initiatives [13]. Despite the increasing international recognition and support for this nursing service, there remains a limited understanding of the full impact of community nursing on health outcomes [14-16].

### The International Classification of Health Interventions

Since its early initiation, ICHI was envisioned as a standardized classification system to describe health care interventions provided by health professionals [17]. To structure the context of this work, developers defined *health intervention* to mean “an act performed for, with or on behalf of a person or a population whose purpose is to assess, improve, maintain, promote or modify health, functioning or health conditions” [18]. The purpose of ICHI was to facilitate the comparison of semantically equivalent information at local, national, or international levels; act as a national classification for countries where no existing (or outdated) intervention classification systems existed; and complement the existing WHO-FIC classifications, ICD and ICF [17,18].

In 2007, working groups within the WHO-FIC began to direct the development of this international classification. A categorial structure, developed by the European Standard Body CEN TC 251/International Standards Organization TC 215 group*,* was used to build and define the included ICHI content including a framework that defined the way concepts would be related to each other [4,17-19].

Semantic categories within ICHI are structured into 3 axes:

- Target: the semantic categories that the intervention (action) is carried out on, to, or with (eg, person, family, and community)
- Action: the semantic categories describing the intervention done by the actor to the target (eg, assessment, treating, assisting, and informing)
- Means: the semantic categories defining the intervention (action) method or process (eg, method, approach, and technique)

In 2012, an alpha version of the classification became available (in Excel format) to affiliated researchers and partners [18]. After several years, iterations, and evaluative projects, the beta version of ICHI became available to the public through a functional Web browser. This browser allowed users to search through over 7000 concepts in 4 category sections [3,4].

1. Interventions on Body Systems and Functions (eg, biomedical body systems)
2. Interventions on Activities and Participation Domains (eg, activities of daily living)
3. Interventions on the Environment (eg, products, services, and systems)
4. Interventions on Health-related Behaviours (eg, safety and lifestyle)

In a recent release of ICHI, developers defined the use of extension codes allowing for the broadening of the intervention classification (eg, assistive and therapeutic products) [4]. This inclusion has allowed for the classification to grow and to continue in relevance [20]. In late 2018, ICHI released a beta-2 version, which included a noted increase in concept coverage and updated resource materials.

### The International Classification for Nursing Practice

The International Council of Nurses (ICN) represents around 20 million nurses in more than 130 nursing associations across the world [21]. ICN develops and distributes ICNP, a standardized terminology system for nursing [22,23]. ICNP conforms to 18104:2014 Health informatics—Categorial structures for representation of nursing diagnoses and nursing actions in terminological systems (previously published as ISO 18104:2003) [24,25]. As a formal standardized nursing terminology, ICNP provides a polyhierarchical framework into which nursing diagnoses, interventions, and outcomes are structured and coded for multiple uses [26].

Since 2005, ICNP has utilized the Web Ontology Language to permit automated description logic reasoning, ensure coherence, and support the classification development [27]. Due to its robustness and compliance to international standards, ICNP is widely recognized as a standard terminology appropriately suited to describe the professional practice of nursing. The WHO has included ICNP as a related classification in the WHO-FIC, using it to extend coverage into the domain of nursing [28].

As an invested partner in the advancement of ICHI, ICN has maintained a working relationship with the ICHI development task force. For example, in 2016, researchers mapped 100 frequently recorded ICNP nursing interventions from acute care settings to the 2015 ICHI alpha release [29]. The purpose was to evaluate the degree of ICNP content coverage in ICHI as well as provide recommendations for additions and changes. The researchers in that study found that 80% of ICNP concepts were represented in ICHI. They also found missing content coverage, ambiguities in concept description, and uncertainties in the semantic matching [30].

## Methods

### Research Design

This is a descriptive research study. The presented work was conducted using a content mapping approach (the most common method used to perform terminology mapping [29,31-35]) in 2 main phases in July and August, 2018. In phase 1, 2 coders completed independent content mapping activities. In phase 2, the 2 coders compared each list and discussed content matches until consensus on ICNP-ICHI match and on mapping relationship was reached. Additional details about these phases are included below.

The community nursing interventions used in this study were derived from the ICNP Community Nursing Catalogue. This catalogue was developed in 2011, updated most recently in 2017, and created in partnership between the Scottish Government and the ICN [36]. The ICN Guidelines for Catalogue Development encourages worldwide validation through global use. The ICNP Community Nursing Catalogue contains 187 community nursing interventions [36]. These interventions (source) were used to identify if there were any equivalent ICHI precoordinated interventions (target) in the draft 2017 beta-1 release.

This study did not require research ethics board review as it had no human subjects or materials and was considered a quality assurance and quality improvement evaluation [37,38].

### Phase 1: Independent Content Mapping

**Figure 1:**
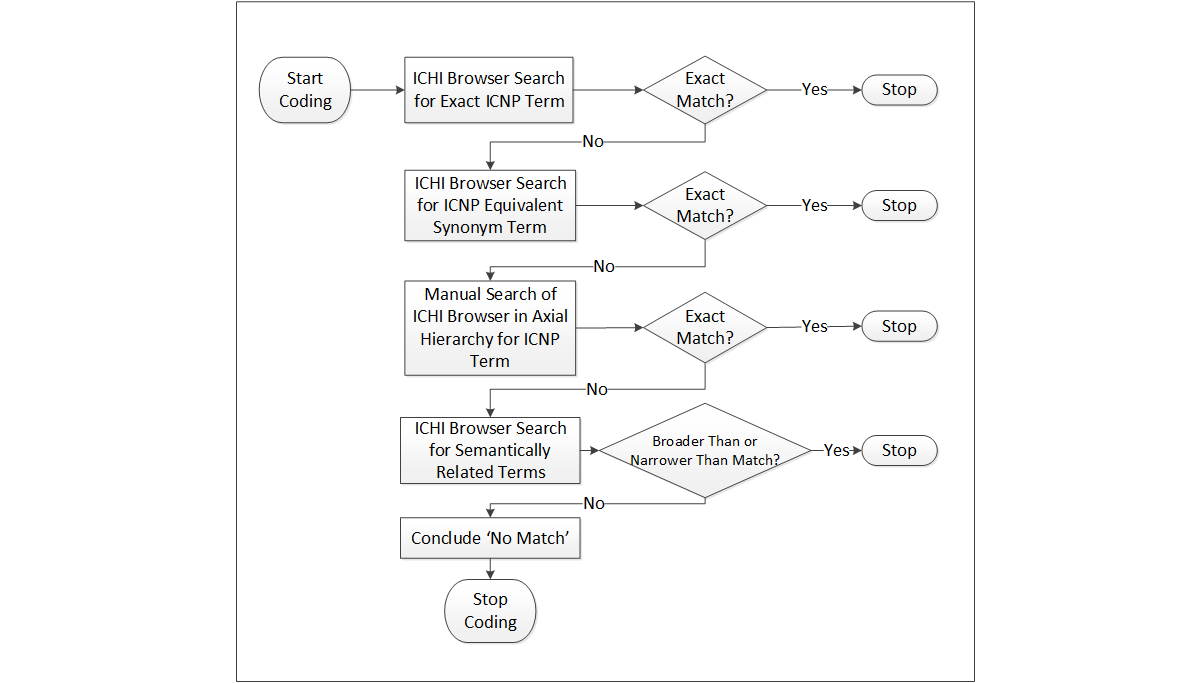
Decision process for mapping International Classification for Nursing Practice (ICNP) to International Classification of Health Interventions (ICHI).

In phase 1, 2 coders (LB, GS) independently mapped 187 ICNP community nursing interventions to ICHI. The mapping process used by each coder to identify a possible ICNP match to an ICHI intervention was completed using the ICHI online browser and followed the method outlined in Figure 1. For example, if exact or equivalent terms were not immediately found in the ICHI browser search bar, the coders manually searched through the axial categories (eg, interventions on body systems and functions), drilling down through the hierarchical layers (eg, interventions on the integumentary system) until a match (or not) was found. These mapping processes facilitated different mechanisms to manage the search of concepts among the thousands of concepts available to view in the ICHI browser. Different mapping relationships were further considered as exact or equivalent (eg, dog-dog), broader than (eg, dog-mammal), or narrower than (eg, dog-Siberian husky) based on their semantic representation.

The coding was performed in batches to ensure consistency in process and to allow the coders to refine the process over time. This was a mechanism that was established to improve the quality and reliability of the mapping process overall. In the first batch, a systematic sampling method was used to mark every 20th ICNP intervention for a total of 10 (n=10) ICNP intervention codes. This small number allowed the coders to refine the mapping process without having a potentially negative influence on the level of agreement calculated at the end of the study. In the second batch, a total of 30 (n=30) different interventions were selected for coding. This number was selected as it allowed for an additional opportunity to include more types of interventions for refinement in the mapping process. Finally, the remaining interventions (n=147) were coded in the final batch. Other members of the team (LC, NH) were regularly consulted throughout this mapping process and acted to ensure the decision process (Figure 1) was maintained. The mapping took place over a period of 2 months (July and August of 2018).

### Phase 2: Reaching Consensus

In phase 2, the independent mapping results were compiled into 1 shared spread sheet. The file contained a list of all ICNP interventions from a particular batch and the matched ICHI intervention from each coder. A percentage agreement between the 2 coders was calculated for each batch. When the coders had different findings from one another, a discussion was carried out until agreement of 1 mapping match was met. The coders also collectively determined the type of mapping relationship for each concept match (equivalent, broader than, narrower than, or no match). These methods are typically followed in content mapping methods to resolve disagreements and come to consensus [29]. As a result, a single ICHI intervention (or no match) was identified for each ICNP intervention. Once completed, final mapping results were presented and discussed among the entire research team, providing opportunity to examine themes and trends of the findings.

## Results

### Phase 1: Independent Content Mapping

In phase 1, independent coding was completed for all of the ICNP interventions. The percentage agreement between the 2 coders was 47% (n=88). There was no agreement between the coders in the remaining cases (n=99). Table 1 shows examples of cases where the coders identified the same ICHI code, where the coders both identified no ICHI code and where there was no initial mapping agreement.

**Table 1:** Phase 1 examples of independent content mapping results.

International Classification for Nursing Practice source term/code	ICHI^a^ term/code by coder #1	ICHI term/code by coder #2	Coding result
10030440 Advising About Employment	SU2.PN.ZZ Advising about work and employment	SU2.PN.ZZ Advising about work and employment	Agreement (map)
10024570 Supporting Caregiver	No ICHI match identified	No ICHI match identified	Agreement (no map)
10031062 Counselling Patient	PZB.PP.ZZ counselling, not elsewhere classified	No ICHI match identified	Disagreement (different ICHI code was identified)

^a^ICHI: International Classification of Health Interventions.

### Phase 2: Reaching Consensus

During phase 2, consensus was achieved for all ICNP interventions (source) through discussion between the 2 coders. A total of 151 cases (81%) of ICNP intervention concepts resulted in an ICHI match. A total of 36 cases (19%) of ICNP intervention concepts resulted in no ICHI match. In the cases where an ICHI match was identified, a conversation ensued about whether ICHI was equivalent to ICNP, whether ICHI was narrower than ICNP, or whether ICHI was broader than ICNP. A summary of the findings and examples are shown in Table 2. Within content mapping methodology, this is a typical approach to identifying equivalency [29,32-35,39].

After the 2 coders completed their mapping consensus work, results were shared with the full research team. As a group, we examined missing ICNP concepts and found thematic groupings that are important practice areas for community nursing. These include intervention concepts related to family and caregivers, death and dying, and case management (Multimedia Appendix 1).

**Table 2:** Summary of mapping results in phase 2 and examples of mapping specificity.

Mapping result	Statistics, N (%)	Example	
		Source: ICNP^a^ intervention term	Target: ICHI^b^ code and term
ICHI was equivalent to ICNP	42 (23)	10030558 Assessing bowel continence	KTK.AA.ZZ Assessment of defecation functions
ICHI was narrower than ICNP	9 (5)	10032994 Teaching about effective parenting	SSK.PM.ZZ Education about parent-child relationships
ICHI was broader than ICNP	100 (53)	10030429 Administering vaccine	DTB.DB.AE Other immunization, not elsewhere classified
No match	36 (19)	10032859 Supporting family coping process	(none found)

^a^ICNP: International Classification for Nursing Practice.

^b^ICHI: International Classification of Health Interventions.

## Discussion

### Principal Findings

The inclusion of community nursing interventions in administrative classifications is essential when evaluating the health and well-being of individuals, families, communities, and populations. The results of this study indicated that 151 of 187 (81%) ICNP community nursing intervention concepts were represented (equivalent, broader, and narrower matches combined) in the beta-1 release of ICHI. Although there is no industry gold standard with which to judge these results, we suggest the representation of community nursing interventions in ICHI appears encouraging. For the 36 (19%) concepts that did not have matches in ICHI, further analysis revealed (1) instances where ICHI was missing representative concepts and (2) inherent differences in terminology system design [29,30]. In addition, the results highlight key considerations related to the representation of knowledge in administrative terminology systems.

### Missing Concept Coverage in International Classification of Health Interventions

A total of 36 ICNP intervention concepts were not represented in the ICHI classification. After examining these missing concepts in greater detail, we were able to thematically group several of the intervention concepts into “family and caregivers,” “death and dying,” and “case management.” Inclusion of concepts in ICHI, which consider these themes, is recommended to ensure related concepts are available for administrative reporting and analysis. A focus on the collection of relevant information about community health care provision is necessary to gain knowledge about general health service provision [9].

It is within the scope of practice for community nurses to care for the families and caregivers of a patient [40-45]. In our sample of 187 community nursing interventions, 10 ICNP concepts related to family or caregivers were not represented in ICHI (ie, *10032859 Supporting Family Coping Process; 10032068 Monitoring For Impaired Family Coping*). In particular, this was noted for those concepts specific to community nursing interventions for caregivers of young children (ie, *10032837 Supporting Caregiver During Weaning; 10033093 Teaching Caregiver About Toilet Training 10032973 Teaching Infant Massage*). This practice is often performed by visiting nurses concerned about the functioning and development of young families. Mapping difficulties were also noted when attempting to match ICNP concepts with the specific word “caregiver,” as ICHI uses different terms in target descriptions (eg, family, friend, peers, colleagues, neighbors, and community members). Although each of these ICHI target terms could be a “caregiver,” in practice, they are not always equivalent. Caring for the caregiver and family is essential to the overall health of a population and necessary to account for in administrative classifications [40-45].

Another area with missing content coverage was noted for those specific ICNP intervention concepts on “death” and “dying” (ie, *10041254 Supporting Dignified Dying; 10033296 Verifying Death*). In the ICHI beta-1 version, no codes specifically used these terms, or even the broader terms of “palliative care,” “hospice,” or “end of life.” This area of practice has always been part of nursing and is increasingly viewed an essential service in the community setting [13]. Cultural, legal, and practice changes are also occurring on this topic of end of life care. For example, in Canada, medical assistance in dying is a legally administered intervention provided by physicians and nurse practitioners and is supported by other health care providers such as registered nurses [46]. Ensuring the representation of appropriate end of life concepts in administrative classifications is necessary as it supports the evaluation of health interventions provided in the community setting.

A theme emerged related to missing content for community nursing “case management”. Case management is the coordination of a wide variety of services, which benefit the care of individuals, families, and communities [13]. For example, the role of community nursing in case management activities may include screening of health and functional needs, arranging services, planning care, ongoing reassessment, and provision of continuity between services [13]. In the report *Crossing the Quality Chasm* [47], the need to improve the organization and coordination of care around the needs of a person was stated as a measure to improve the health care system. Though the mapping between ICNP and ICHI did find matches between related concepts (ie, *10030455 Advising About Housing*), several were not found (ie, *10032598 Referring To Housing Service; 10030625 Assessing Housing Condition; 10030493 Arranging Transport of Device*). These missing concepts describe the type of ongoing case management community nurses provide on behalf the persons outside of institutionalized care. It is again recommended that case management intervention concepts continue to be developed and added to administrative classification systems as a means to increase our understanding and inform future health care decisions.

### Foundational Design Decisions of a Classification System

The foundational design of a classification or terminology system considers scope, hierarchical orientation, concept granularity, and concept placement. Standards such as ISO 18104:2014 Health informatics--Categorial structures for representation of nursing diagnoses and nursing actions in terminological systems, direct design decisions. For example, ICHI concepts are required to include a defined target, action, and means. ICNP interventions are required to have a target and action but no means. When researchers conduct interterminology mapping exercises, discord between concept representations may be related to these foundational development decisions.

In this mapping activity, several missing ICNP matches were related to differences in concept granularity (ie, specificity or level of detail for related concept). For example, the ICNP concept *10033126 Teaching Patient* was determined to have “no match” in ICHI. This was not because of the lack of codes in ICHI, which could be used to describe patient education. Rather, the ICNP concept was “broader than” what was available in ICHI. One may then ask, why not choose an ICHI concept that was more specific and call it a “narrower match?” The ICNP concept *10033126 Teaching Patient* could have been a “narrower match” to over 300-specific ICHI educational concepts. Practically speaking, the terminology coders could not make a meaningful one-to-one match. The following examples represent additional “broader than” ICNP concepts that did not have meaningful matches in ICHI.

- 10030673 Assessing During Encounter
- 10024570 Supporting Caregiver
- 10032844 Supporting Family
- 10031912 Managing Disease
- 10031965 Managing Symptom
- 10033086 Teaching Caregiver
- 10033126 Teaching Patient

This example highlights the complexities of knowledge representation when attempting to map terminologies of varying granularity and overlapping coverage. When decisions are made on how a terminology or classification is to be foundationally structured, and then mapped to another with a different foundational base, clashes in semantic matching may be part of the expected results.

### Representation of Community Nursing Practice

As noted above, a total of 151 (81%) ICNP community nursing interventions are represented in ICHI. Two-thirds of these concept matches were classified as “broader than” (ie, meaning that an ICNP concept could fit as a “child” into the broader ICHI “parent” concept). From the vantage of developing an administrative classification to represent health, it can be understood that there has to be a threshold of low specificity to allow for a higher aggregation of data. However, the question remains as to whether these “broader than” ICHI concepts satisfactorily represent nursing care interventions and at what point knowledge representation turns from meaningful coverage to diluted meaninglessness.

**Table 3:** International Classification for Nursing Practice concepts not elsewhere classified.

International Classification for Nursing Practice concept	“Broader than” International Classification of Health Interventions concept
10031117 Diabetic Ulcer Care	LZZ.ZY.ZZ Other interventions on integumentary system, not elsewhere classified
10031690 Malignant Wound Care	LZZ.ZY.ZZ Other interventions on integumentary system, not elsewhere classified
10032420 Pressure Ulcer Care	LZZ.ZY.ZZ Other interventions on integumentary system, not elsewhere classified
10032863 Surgical Wound Care	LZZ.ZY.ZZ Other interventions on integumentary system, not elsewhere classified
10033208 Traumatic Wound Care	LZZ.ZY.ZZ Other interventions on integumentary system, not elsewhere classified
10033254 Ulcer Care	LZZ.ZY.ZZ Other interventions on integumentary system, not elsewhere classified
10030710 Assessing Risk For Pressure Ulcer	LZZ.ZY.ZZ Other interventions on integumentary system, not elsewhere classified
10030723 Assessing Risk For Transfer Injury	LZZ.ZY.ZZ Other interventions on integumentary system, not elsewhere classified
10031931 Managing Postpartum Care	NUE.ZY.ZZ Other interventions on functions related to pregnancy, not elsewhere classified
10031949 Managing Prenatal Care	NUE.ZY.ZZ Other interventions on functions related to pregnancy, not elsewhere classified
10030706 Assessing Risk For Depressed Mood During Postpartum Period	NUE.ZY.ZZ Other interventions on functions related to pregnancy, not elsewhere classified
10031769 Managing Postpartum Depressed Mood	NUE.ZY.ZZ Other interventions on functions related to pregnancy, not elsewhere classified
10031805 Managing Enuresis	NTD.ZY.ZZ Other interventions on urination function, not elsewhere classified
10031879 Managing Urinary Incontinence	NTD.ZY.ZZ Other interventions on urination function, not elsewhere classified
10033135 Teaching Self-Catheterisation	NTD.ZY.ZZ Other interventions on urination function, not elsewhere classified
10033277 Urinary Catheter Care	NTD.ZY.ZZ Other interventions on urination function, not elsewhere classified
10035958 Facilitating Grief	AUD.ZY.ZZ Other interventions on emotional functions, not elsewhere classified
10031711 Managing Anxiety	AUD.ZY.ZZ Other interventions on emotional functions, not elsewhere classified
10031851 Managing Negative Emotion	AUD.ZY.ZZ Other interventions on emotional functions, not elsewhere classified

In the case of community nursing skin and wound care concepts, 90% were matched to ICHI as “broader than” (10% no matches). For example, 8 skin and wound care ICNP concepts were rolled up into the closest ICHI match, *LZZ.ZY.ZZ Other interventions on integumentary system, not elsewhere classified*. Similar outcomes were found for ICNP concepts related to prenatal and postpartum care, continence and catheter care, and supporting care for grief and anxiety (Table 3). If these concepts were subsequently mapped against health care data, the knowledge represented would be so vague that extracting knowledge back out of it could be lost. These are important considerations, especially as these concepts not only represent the care provided by community nursing but also many other health care professional groups. ICHI is being developed for countries to report and analyze on health interventions [3]. It is recommended, therefore, that ongoing work continues to evaluate the practical use (eg, to support resourcing) of those concept groups frequently mapped as “broader than,” to ensure the meaningful representation of health care phenomena is available in administrative classifications [20].

### Mapping Methods of Coding and Consensus

There is no agreed upon method of mapping concepts from a source to a target classification or terminology. Multiple examples of mapping clinical content between interterminology groups, datasets to terminologies, or raw clinical content exist [23,29,39,48,49]. In this study, we presented a method of using 2 coders to manually map 187 concepts from 1 standardized clinical terminology to another standardized clinical classification. This mapping exercise was greatly aided by both the ICHI and ICNP publicly available Web browsers.

During phase 1, only 47% (n=88) of the concept matches were the same between the 2 coders; this increased to 100% in phase 2. Although the percentage agreement was low at the beginning, statistically suggesting weakness in the initial findings [50], the science of clinical informatics is still maturing and has yet to demonstrate how this value fully impacts the reliability of mapping results [51]. It is possible that this lower agreement was related to large number of target concepts (eg, ICHI beta-1 version had 7000 concepts), differences in concept understanding (eg, differences between counseling, advising, education, and emotional support), and different levels of experience in mapping ICNP and ICHI content.

To increase the trustworthiness of the content mapping process, batches of coding and consensus gathering were completed to provide a quality assurance mechanism by allowing the coders to further clarify and consistently manage the coding process. During the first batch of intervention discussions, senior researchers in field (LC, NH) provided coaching regarding how to consistently manage the coding process. This acted as a quality control mechanism before the remainder of the content mapping was completed. The remaining batches were discussed and resolved without the senior researchers’ presence. The browser tool was also used throughout the consensus discussions between the 2 coders. In particular, when debating between 2 different ICHI concepts, the coders would consult the concept definition and inclusion fields found when clicking the ICHI concept. This discussion process facilitated a final 100% agreement of mapping results by the end of phase 2.

Finally, it should be noted that the coders are registered nurses with both clinical practice and content mapping experience. This facilitated the coders to use explicit knowledge to understand concept meaning in context to community care, to find concept synonyms (eg, step 2 in the mapping method process), and to easily navigate the ICHI Web browser. It is outside the scope of this study to examine how tacit knowledge, experiential judgment, or social relationships (eg, consensus agreement) may have contributed to the coders’ mapping choices. Future researchers may wish to examine the influence of these variables on concept terminology mapping results. For example, those research methods that capture the decision-making process of a coding task (eg, Think Aloud protocols) may potentially be a fruitful line of inquiry.

### Limitations

There are limitations related to the repeatability of this study. Though we have attempted to be clear and robust in the methods and processes used to map the ICNP community content to ICHI, the findings may have been different had there been different coders or different versions of classifications. For example, the ICHI beta-1 version (utilized over the coding period of Summer 2018) was updated in October 2018 to ICHI beta-2, increasing clinical concepts from approximately 7000 to 8000. It is possible that the rate of agreement between the 2 classifications would be different with updated and ongoing versions.

### Conclusions

The collection of standardized information from electronic health records is used to help institutions to determine priorities and effective allocation of resources [10]. As the shift toward preventative and community-based health care increases, so too does the need for health organizations to have well informed administrative data about this domain. The work presented in this study helps advance the representation of community nursing concepts in administrative datasets, a relatively new challenge for nursing informatics; however, although this is a necessary step, it does not guarantee that these data will be utilized in reporting. Continued work is necessary to champion and value the work of community nursing, which will further contribute to a wholesome account of the health and well-being of individual, families, communities, and populations.

## Appendix

Multimedia Appendix 1 International Classification for Nursing Practice to International Classification of Health Interventions community nursing mapping results.

## Abbreviations

ICD: International Classification of Diseases
ICF: International Classification of Functioning, Disability and Health
ICHI: International Classification of Health Interventions
ICNP: International Classification for Nursing Practice
WHO-FIC: World Health Organization Family of International Classifications
