# Supplementary material for: Visibility of Community Nursing Within an Administrative Health Classification System: Evaluation of Content Coverage
Source: J Med Internet Res. 2019 Jun 26;21(6):e12847. doi: 10.2196/12847 (PMC6617914; doi:10.2196/12847)
Supplement: Multimedia Appendix 1 [file jmir_v21i6e12847_app1.pdf]

Multimedia Appendix 1: ICNP to ICHI Community Nursing Mapping Results

ICNP Intervention Concept	ICHI_Code	ICHI Concept	Matching(picklist)
10030687 Admission Assessment	PZA.AE.AH	Physical examination, whole body	Narrower--ICHI is narrower than ICNP
10030673 Assessing During Encounter			No match-ICHI has no match to ICNP term
10030618 Assessing Health And Social Care Needs			No match-ICHI has no match to ICNP term
10030558 Assessing Bowel Continence	KTK.AA.ZZ	Assessment of defecation functions	Exact match -ICHI is equivalent to ICNP
10030781 Assessing Urinary Continence	NTD.AA.ZZ	Assessment of urination functions	Broader-ICHI is broader than ICNP
10030884 Catheterising Urinary Bladder	NAI.JB.AC	Drainage of bladder without incision	Broader-ICHI is broader than ICNP
10041427 Managing Defaecation	KTK.RB.ZZ	Practical support with defaecation function	Broader-ICHI is broader than ICNP
10031782 Managing Encopresis	KTK.ZY.ZZ	Other interventions on defaecation function, not elsewhere classified	Broader-ICHI is broader than ICNP
10031805 Managing Enuresis	NTD.ZY.ZZ	Other interventions on urination function, not elsewhere classified	Broader-ICHI is broader than ICNP
10031879 Managing Urinary Incontinence	NTD.ZY.ZZ	Other interventions on urination function, not elsewhere classified	Broader-ICHI is broader than ICNP
10032150 Nephrostomy Care			No match-ICHI has no match to ICNP term
10032788 Stoma Care	PTD.ZY.ZZ	Other stoma care, not elsewhere classified	Exact match -ICHI is equivalent to ICNP
10032987 Teaching About Nephrostomy Care			No match-ICHI has no match to ICNP term
10033055 Teaching About Stoma Care	PTD.PM.ZZ	Stoma education	Exact match -ICHI is equivalent to ICNP
10033135 Teaching Self-Catheterisation	NTD.ZY.ZZ	Other interventions on urination function, not elsewhere classified	Broader-ICHI is broader than ICNP
10033277 Urinary Catheter Care	NTD.ZY.ZZ	Other interventions on urination function, not elsewhere classified	Exact match -ICHI is equivalent to ICNP
10030809 Assisting Health Care Provider			No match-ICHI has no match to ICNP term
10030911 Checking Patient Identity			No match-ICHI has no match to ICNP term

10030953 Collaborating With Legal Service	PZB.TA.ZZ	Advocating for a person	Broader-ICHI is broader than ICNP
10005029 Consulting Health Care Provider	PZB.TD.ZZ	Case coordination	Exact match -ICHI is equivalent to ICNP
10031027 Coordinating Care Plan	PZB.TB.ZZ	Individualised planning	Exact match -ICHI is equivalent to ICNP
10035825 Crisis Management	UE1.VA.ZZ	Building the capacity of services, systems and policies, in preparedness for disasters and emergencies that may impact on health	Narrower--ICHI is narrower than ICNP
10006016 Discharge Planning	PZB.TB.ZZ	Individualised planning	Exact match -ICHI is equivalent to ICNP
10031252 Evaluating Care Plan	PZB.TB.ZZ	Individualised planning	Broader-ICHI is broader than ICNP
10031822 Managing Health Status After Hospitalisation	PZB.ZY.ZZ	Other health management encounter, not elsewhere classified	Broader-ICHI is broader than ICNP
10031867 Managing Negative Response To Situation	AS1.ZY.ZZ	Other interventions on mental functions	Narrower-ICHI is narrower than ICNP
10032567 Referring to Health Care Provider	PZB.TC.ZZ	Navigating the service system	Broader-ICHI is broader than ICNP
10041254 Supporting Dignified Dying			No match-ICHI has no match to ICNP term
10033296 Verifying Death			No match-ICHI has no match to ICNP term
10030562 Assessing Caregiver	UC1.AA.ZZ	Assessment of support and relationships	Narrower-ICHI is narrower than ICNP
10024570 Supporting Caregiver			No match-ICHI has no match to ICNP term
10030589 Assessing Emotional Support	UC1.AA.ZZ	Assessment of support and relationships	Broader-ICHI is broader than ICNP
10030813 Assisting With Anger Control	SDJ.RC.ZZ	Emotional support for managing stress and demands	Broader-ICHI is broader than ICNP
10031062 Counselling Patient	PZB.PP.ZZ	Counselling, not elsewhere classified	Exact match -ICHI is equivalent to ICNP
10035958 Facilitating Grief	AUD.ZY.ZZ	Other interventions on emotional functions, not elsewhere classified	Broader-ICHI is broader than ICNP
10031711 Managing Anxiety	AUD.ZY.ZZ	Other interventions on emotional functions, not elsewhere classified	Broader-ICHI is broader than ICNP
10031833 Managing Negative Behaviour	VEL.ZY.ZZ	Other interventions targeting behaviours related to psychological health and wellbeing, not elsewhere classified	Broader-ICHI is broader than ICNP
10031851 Managing Negative Emotion	AUD.ZY.ZZ	Other interventions on emotional functions, not elsewhere classified	Broader-ICHI is broader than ICNP

10032505 Promoting Psychological Status	VEL.PN.ZZ	Advising about behaviours related to psychological health and wellbeing	Exact match -ICHI is equivalent to ICNP
10027051 Providing Emotional Support	SMH.RC.ZZ	Emotional support for looking after one's health	Broader-ICHI is broader than ICNP
10024493 Providing Instructional Material	VFX.ZY.ZZ	Other interventions targeting other health-related behaviours, not elsewhere classified	Broader-ICHI is broader than ICNP
10027067 Providing Spiritual Support	SXH.RB.ZZ	Practical support with engagement in religion and spirituality	Exact match -ICHI is equivalent to ICNP
10030493 Arranging Transport Of Device			No match-ICHI has no match to ICNP term
10033368 Assessing Need	PZB.TB.ZZ	Individualised planning	Broader-ICHI is broader than ICNP
10030924 Checking Device For Safety	UA1.AA.ZZ	Assessment of products and technology	Broader-ICHI is broader than ICNP
10031776 Managing Device	UA1.SM.ZZ	Management of products and technology	Exact match -ICHI is equivalent to ICNP
10032902 Teaching About Device	UA1.PM.ZZ	Education about products and technology	Exact match -ICHI is equivalent to ICNP
10030440 Advising about Employment	SU2.PN.ZZ	Advising about work and employment	Exact match -ICHI is equivalent to ICNP
10030602 Assessing Family Process	SSJ.AA.ZZ	Assessment of family relationships	Exact match -ICHI is equivalent to ICNP
10044963 Intimate Partner Violence Therapy	VBB.ZY.ZZ	Other intervention targeting family and partner violence behaviours, not elsewhere classified	Broader-ICHI is broader than ICNP
10032844 Supporting Family			No match-ICHI has no match to ICNP term
10032859 Supporting Family Coping Process			No match-ICHI has no match to ICNP term
10032994 Teaching about Effective Parenting	SSK.PM.ZZ	Education about parent-child relationships	Narrower-ICHI is narrower than ICNP
10030429 Administering Vaccine	DTB.DB.AE	Other immunisation, not elsewhere classified	Broader-ICHI is broader than ICNP
10030969 Collecting Cervical Cells	NMF.AH.AC	Cervical papanicolaou smear	Narrower-ICHI is narrower than ICNP
10031036 Counselling about Alcohol Use	VAA.PP.ZZ	Counselling about alcohol use behaviours	Exact match -ICHI is equivalent to ICNP
10031043 Counselling about Drug Abuse	VAC.PP.ZZ	Counselling about illicit drug use behaviours	Exact match -ICHI is equivalent to ICNP
10031058 Counselling about Tobacco Use	VAB.PM.ZZ	Education to influence tobacco use behaviours	Exact match -ICHI is equivalent to ICNP
10032465 Promoting Health Seeking Behaviour	VDC.VB.ZZ	Awareness raising to influence health service use behaviuors	Exact match -ICHI is equivalent to ICNP

10032477 Promoting Hygiene	VED.VB.ZZ	Awareness raising to influence hygiene behaviours	Exact match -ICHI is equivalent to ICNP
10032483 Promoting Oral Hygiene	VEE.VB.ZZ	Awareness raising to influence oral hygiene behaviours	Exact match -ICHI is equivalent to ICNP
10041086 Smoking Cessation Therapy	VAB.PP.ZZ	Counselling about tobacco use behaviours	Narrower-ICHI is narrower than ICNP
10032580 Referring To Family Planning Service	PZB.TC.ZZ	Navigating the service system	Broader-ICHI is broader than ICNP
10032726 Screening Patient			No match-ICHI has no match to ICNP term
10033017 Teaching About Rehabilitation	UEP.PM.ZZ	Education about health services, systems and policies	Broader-ICHI is broader than ICNP
10033001 Teaching About Effective Weight	KTN.PM.ZZ	Education about weight maintenance	Exact match -ICHI is equivalent to ICNP
10040125 Teaching About Exercise	VEB.PM.ZZ	Education to influence physical activity behaviours	Exact match -ICHI is equivalent to ICNP
10032925 Teaching About Family Planning	NUC.PM.ZZ	Education about procreation functions	Broader-ICHI is broader than ICNP
10032941 Teaching About Head Lice Infestation	VED.PM.ZZ	Education to influence hygiene practices	Broader-ICHI is broader than ICNP
10033038 Teaching About Sexual Behaviour	VEF.PM.ZZ	Education to influence sexual behaviours	Exact match -ICHI is equivalent to ICNP
10033064 Teaching About Travel Health			No match-ICHI has no match to ICNP term
10033072 Teaching About Vaccine	VDA.PM.ZZ	Education to influence immunisation behaviours	Broader-ICHI is broader than ICNP
10024687 Teaching Safety Measures	VCD.PM.ZZ	Education to influence other safety behaviours	Broader-ICHI is broader than ICNP
10030515 Assessing Ability To Communicate By Talking	SE1.AA.ZZ	Assessment of communication	Broader-ICHI is broader than ICNP
10030570 Assessing Child Development	ETM.AA.ZZ	Assessment of growth and development	Broader-ICHI is broader than ICNP
10031559 Implementing Immunisation Schedule	VDA.ZY.ZZ	Other interventions targeting immunisation behaviours, not elsewhere classified	Broader-ICHI is broader than ICNP
10031983 Massaging Infant	MRS.PC.ZZ	Massage of whole body	Broader-ICHI is broader than ICNP
10032081 Monitoring Height	PZA.AB.ZZ	Body measurement of whole body	Broader-ICHI is broader than ICNP
10032121 Monitoring Weight	PZA.AB.ZZ	Body measurement of whole body	Broader-ICHI is broader than ICNP
10032454 Promoting Child Development	ETM.PM.ZZ	Education about growth and development	Broader-ICHI is broader than ICNP
10032533 Providing Health Promotion Service For Child Development			No match-ICHI has no match to ICNP term

10032695 Screening Child Development	ETM.AA.ZZ	Assessment of growth and development	Broader-ICHI is broader than ICNP
10032719 Screening Infant Development	ETM.AA.ZZ	Assessment of growth and development	Broader-ICHI is broader than ICNP
10032703 Screening Hearing	CTB.AA.ZZ	Assessment of hearing functions	Exact match -ICHI is equivalent to ICNP
10032735 Screening Vision	BTB.AA.ZZ	Assessment of seeing functions	Exact match -ICHI is equivalent to ICNP
10032816 Supporting Breastfeeding	VEH.RB.ZZ	Practical support with breastfeeding	Exact match -ICHI is equivalent to ICNP
10032828 Supporting Caregiver During Toilet Training			No match-ICHI has no match to ICNP term
10032837 Supporting Caregiver During Weaning			No match-ICHI has no match to ICNP term
10032918 Teaching About Eating Pattern	VEA.PM.ZZ	Education to influence eating behaviours	Broader-ICHI is broader than ICNP
10040380 Teaching About Sleep	VEC.PM.ZZ	Education to influence sleep and rest behaviours	Exact match -ICHI is equivalent to ICNP
10033093 Teaching Caregiver About Toilet Training			No match-ICHI has no match to ICNP term
10033103 Teaching Caregiver About Weaning			No match-ICHI has no match to ICNP term
10032973 Teaching Infant Massage			No match-ICHI has no match to ICNP term
10030417 Administering Insulin	PZX.DB.AZ	Administering pharmacotherapy, not elsewhere classified	Broader-ICHI is broader than ICNP
10030438 Administering Vitamin B12	PZX.DB.AZ	Administering pharmacotherapy, not elsewhere classified	Broader-ICHI is broader than ICNP
10039284 Anticoagulation Therapy	PZX.DB.AZ	Administering pharmacotherapy, not elsewhere classified	Broader-ICHI is broader than ICNP
10030907 Checking Inhalation Technique			No match-ICHI has no match to ICNP term
10031268 Evaluating Treatment Regime	PZB.TB.ZZ	Individualised planning	Broader-ICHI is broader than ICNP
10031674 Maintain Airway	JTB.ZY.ZZ	Other interventions on respiration function, not elsewhere classified	Exact match -ICHI is equivalent to ICNP
10031912 Managing Disease			No match-ICHI has no match to ICNP term
10032034 Monitoring Blood Glucose	DIA.AB.AF	Measurement of blood properties	Broader-ICHI is broader than ICNP
10032052 Monitoring Blood Pressure	HT2.AA.ZZ	Assessment of cardiovascular function	Broader-ICHI is broader than ICNP
10032099 Monitoring Laboratory Results			No match-ICHI has no match to ICNP term
10039369 Oxygen Therapy	PZA.DD.AC	Oxygen therapy	Exact match -ICHI is equivalent to ICNP

10026347 Promoting Self-Care	SM1.TA.ZZ	Advocacy in relation to self care	Exact match -ICHI is equivalent to ICNP
10032800 Supporting Ability To Managing Regime			No match-ICHI has no match to ICNP term
10033161 Tracheostomy Care	PTC.AA.ZZ	Tracheostomy assessment	Narrower--ICHI is narrower than ICNP
10046579 Administering Inhalent Medication	PZX.DB.AC	Administering pharmacotherapy, per orifice	Broader-ICHI is broader than ICNP
10045827 Administering Intramuscular Medication	PZX.DB.AE	Administering pharmacotherapy, percutaneous transparietal	Broader-ICHI is broader than ICNP
10045836 Administering Intravenous Medication	PZX.DB.AF	Administering pharmacotherapy, percutaneous transluminal	Broader-ICHI is broader than ICNP
10025444 Administering Medication	PZX.DB.AZ	Administering pharmacotherapy, not elsewhere classified	Exact match -ICHI is equivalent to ICNP
10045843 Administering Subcutaneous Medication	PZX.DB.AE	Administering pharmacotherapy, percutaneous transparietal	Broader-ICHI is broader than ICNP
10045858 Administering Vaginal Medication	PZX.DB.AC	Administering pharmacotherapy, per orifice	Broader-ICHI is broader than ICNP
10023888 Managing Medication Regime			No match-ICHI has no match to ICNP term
10015523 Prescribing Medication	PZX.TI.ZZ	Pharmacotherapy prescription	Exact match -ICHI is equivalent to ICNP
10030527 Assessing Mobility	SH1.AA.ZZ	Assessment of mobility	Exact match -ICHI is equivalent to ICNP
10033188 Transferring Patient	SHG.RA.ZZ	Performing the task of transferring the person	Narrower-ICHI is narrower than ICNP
10030660 Assessing Nutritional Status	KTE.AA.ZZ	Assessment of digestive functions	Broader-ICHI is broader than ICNP
10039330 Fluid Therapy	PZZ.ZY.ZZ	Other interventions on other and unspecified body systems and functions, not elsewhere classified	Broader-ICHI is broader than ICNP
10031795 Managing Enteral Feeding	PZA.DA.AC	Intracavity administration of nutritional substance	Broader-ICHI is broader than ICNP
10031908 Managing Parenteral Feeding	PZA.DA.AF	Intravenous administration of nutritional substance	Broader-ICHI is broader than ICNP
10046533 Teaching about Diet	VEA.PM.ZZ	Education to influence eating behaviours	Exact match -ICHI is equivalent to ICNP
10032939 Teaching about Fluid Intake	ETE.PM.ZZ	Education about water, mineral and electrolyte balance function	Broader-ICHI is broader than ICNP
10030821 Assisting With Hygiene	VED.ZY.ZZ	Other interventions targeting hygiene behaviours, not elsewhere classified	Broader-ICHI is broader than ICNP

10023531 Assisting With Toileting	SMD.RB.ZZ	Practical support with toileting	Exact match -ICHI is equivalent to ICNP
10031164 Dressing Patient	SME.RA.ZZ	Dressing the person	Exact match -ICHI is equivalent to ICNP
10031275 Eye Care	BZZ.ZY.AZ	Other interventions on eye, not elsewhere classified	Broader-ICHI is broader than ICNP
10032184 Oral Care	VEE.ZY.ZZ	Other interventions targeting oral hygiene behaviours, not elsewhere classified	Broader-ICHI is broader than ICNP
10032757 Skin Care	LT2.ZY.ZZ	Other interventions to functions of the skin, not elsewhere classified	Broader-ICHI is broader than ICNP
10031931 Managing Postpartum Care	NUE.ZY.ZZ	Other interventions on functions related to pregnancy, not elsewhere classified	Broader-ICHI is broader than ICNP
10031949 Managing Prenatal Care	NUE.ZY.ZZ	Other interventions on functions related to pregnancy, not elsewhere classified	Broader-ICHI is broader than ICNP
10032496 Promoting Effective Parenting	VEJ.PM.ZZ	Education to influence parenting behaviours	Broader-ICHI is broader than ICNP
10032885 Teaching About Breast Care During Postpartum Period	NUE.PM.ZZ	Antenatal or postnatal education	Broader-ICHI is broader than ICNP
10032892 Teaching About Breast Care During Prenatal Period	NUE.PM.ZZ	Antenatal or postnatal education	Broader-ICHI is broader than ICNP
10030543 Assessing Arterial Blood Flow Using Ultrasound	ITB.AA.ZZ	Assessment of blood vessel functions	Broader-ICHI is broader than ICNP
10030775 Assessing Tissue Perfusion	ITB.AA.ZZ	Assessment of blood vessel functions	Broader-ICHI is broader than ICNP
10030656 Assessing Urinary Retention Using Ultrasound	NTD.AA.ZZ	Assessment of urination functions	Broader-ICHI is broader than ICNP
10002866 Assisting Surgeon During Operation			No match-ICHI has no match to ICNP term
10004588 Collecting Specimen	PZX.AH.AZ	Specimen collection, not elsewhere classified	Exact match -ICHI is equivalent to ICNP
10031140 Diagnostic Testing			No match-ICHI has no match to ICNP term
10031332 Flushing Earwax	CZZ.JA.AC	Irrigation of ear, not elsewhere classified	Exact match -ICHI is equivalent to ICNP
10031724 Managing Central Line	IZZ.SN.AF	Management of vascular device	Exact match -ICHI is equivalent to ICNP
10032006 Measuring Body Temperature	ETG.AB.ZZ	Measuring body temperature	Exact match -ICHI is equivalent to ICNP
10032113 Monitoring Vital Signs			No match-ICHI has no match to ICNP term
10032047 Monitoring Blood Oxygen Saturation Using Pulse Oximeter	DIA.AB.AF	Measurement of blood properties	Broader-ICHI is broader than ICNP

10032258 Physical Examination	PZA.AE.AH	Physical examination, whole body	Exact match -ICHI is equivalent to ICNP
10033220 Treating Injury	PZB.ZY.ZZ	Other health management encounter, not elsewhere classified	Broader-ICHI is broader than ICNP
10033323 Weighing Patient	PZA.AB.ZZ	Body measurement of whole body	Broader-ICHI is broader than ICNP
10023520 Assessing Risk For Falls	SH2.AA.ZZ	Assessment of changing and maintaining body position	Broader-ICHI is broader than ICNP
10030706 Assessing Risk For Depressed Mood During Postpartum Period	NUE.ZY.ZZ	Other interventions on functions related to pregnancy, not elsewhere classified	Broader-ICHI is broader than ICNP
10030710 Assessing Risk For Pressure Ulcer	LZZ.ZY.ZZ	Other interventions on integumentary system, not elsewhere classified	Broader-ICHI is broader than ICNP
10030723 Assessing Risk For Transfer Injury	LZZ.ZY.ZZ	Other interventions on integumentary system, not elsewhere classified	Broader-ICHI is broader than ICNP
10031846 Managing Impaired Coping Process	SDJ.RB.ZZ	Practical support with handling demands and stress	Broader-ICHI is broader than ICNP
10031769 Managing Postpartum Depressed Mood	NUE.ZY.ZZ	Other interventions on functions related to pregnancy, not elsewhere classified	Broader-ICHI is broader than ICNP
10032075 Monitoring For Child Abuse	VBB.AA.ZZ	Assessment of family and partner violence behaviours	Broader-ICHI is broader than ICNP
10032068 Monitoring For Impaired Family Coping			No match-ICHI has no match to ICNP term
10032960 Teaching About House Safety	VCC.PM.ZZ	Education to influence home safety behaviours	Exact match -ICHI is equivalent to ICNP
10030472 Applying Compression Bandage	LZZ.DK.ZZ	Application of dressing to skin or subcutaneous cell tissue, not elsewhere classified	Broader-ICHI is broader than ICNP
10030486 Applying Elastic Stockings	PZX.LL.AH	Application of pressure garment	Broader-ICHI is broader than ICNP
10030747 Assessing Self Care Of Skin	SMC.AA.ZZ	Assessment of caring for body parts	Broader-ICHI is broader than ICNP
10030799 Assessing Wound	LZZ.AA.ZZ	Assessment of skin and subcutaneous cell tissue, not elsewhere classified	Broader-ICHI is broader than ICNP
10031117 Diabetic Ulcer Care	LZZ.ZY.ZZ	Other interventions on integumentary system, not elsewhere classified	Broader-ICHI is broader than ICNP
10035147 Compression Therapy			No match-ICHI has no match to ICNP term
10031592 Invasive Device Site Care			No match-ICHI has no match to ICNP term

10031690 Malignant Wound Care	LZZ.ZY.ZZ	Other interventions on integumentary system, not elsewhere classified	Broader-ICHI is broader than ICNP
10032420 Pressure Ulcer Care	LZZ.ZY.ZZ	Other interventions on integumentary system, not elsewhere classified	Broader-ICHI is broader than ICNP
10040224 Pressure Ulcer Prevention	LT2.ZY.ZZ	Other interventions to functions of the skin, not elsewhere classified	Broader-ICHI is broader than ICNP
10032630 Removing Suture	LZZ.JD.AA	Removal of foreign body from skin and subcutaneous cell tissue, site not specified	Broader-ICHI is broader than ICNP
10032648 Removing Wound Clip	LZZ.JD.AA	Removal of foreign body from skin and subcutaneous cell tissue, site not specified	Broader-ICHI is broader than ICNP
10032863 Surgical Wound Care	LZZ.ZY.ZZ	Other interventions on integumentary system, not elsewhere classified	Broader-ICHI is broader than ICNP
10032871 Suture Wound	LZZ.MK.AA	Other repair of skin and subcutaneous cell tissue, site not specified	Broader-ICHI is broader than ICNP
10033029 Teaching About Self Care Of Skin	SMC.PM.ZZ	Education about caring for body parts	Broader-ICHI is broader than ICNP
10034961 Teaching About Wound Care	LZZ.PM.ZZ	Education about skin and subcutaneous cell tissue, not elsewhere classified	Broader-ICHI is broader than ICNP
10034974 Teaching About Wound Healing	LZZ.PM.ZZ	Education about skin and subcutaneous cell tissue, not elsewhere classified	Broader-ICHI is broader than ICNP
10033208 Traumatic Wound Care	LZZ.ZY.ZZ	Other interventions on integumentary system, not elsewhere classified	Broader-ICHI is broader than ICNP
10033231 Treating Skin Condition	LT2.ZY.ZZ	Other interventions to functions of the skin, not elsewhere classified	Broader-ICHI is broader than ICNP
10033249 Wart Treatment	LZZ.GA.AA	Destruction of lesion or tissue of skin and subcutaneous cell tissue, site not specified	Broader-ICHI is broader than ICNP
10033254 Ulcer Care	LZZ.ZY.ZZ	Other interventions on integumentary system, not elsewhere classified	Broader-ICHI is broader than ICNP
10030455 Advising About Housing	UEE.PN.ZZ	Advising about housing services, systems and policies	Broader-ICHI is broader than ICNP
10030536 Assessing Ability To Prepare Food	SO2.AA.ZZ	Assessment of household task performance	Broader-ICHI is broader than ICNP
10030625 Assessing Housing Condition			No match-ICHI has no match to ICNP term

10024298 Assessing Social Support	UEO.AA.ZZ	Assessment of general social support services, systems and policies	Broader-ICHI is broader than ICNP
10032598 Referring To Housing Service			No match-ICHI has no match to ICNP term
10011660 Managing Pain	AXA.ZY.ZZ	Other interventions for pain, not elsewhere classified	Broader-ICHI is broader than ICNP
10031965 Managing Symptom			No match-ICHI has no match to ICNP term
10033086 Teaching Caregiver			No match-ICHI has no match to ICNP term
10033126 Teaching Patient			No match-ICHI has no match to ICNP term
